# Progressive worsening of aortic regurgitation due to detachment of the aortic valve commissure with multimodality imaging to elucidate pathogenesis: a case report

**DOI:** 10.1093/ehjcr/ytae178

**Published:** 2024-04-09

**Authors:** Shun Nishino, Masanori Nishimura, Yujiro Asada, Atsushi Yamashita, Yoshisato Shibata

**Affiliations:** Department of Cardiology, Miyazaki Medical Association Hospital Cardiovascular Center, 1173 Arita, Miyazaki 880-2102, Japan; Department of Cardiothoracic Surgery, Miyazaki Medical Association Hospital Cardiovascular Center, Miyazaki, Japan; Department of Pathology, Miyazaki Medical Association Hospital Cardiovascular Center, Miyazaki, Japan; Department of Pathology, Faculty of Medicine, University of Miyazaki, Miyazaki, Japan; Department of Cardiology, Miyazaki Medical Association Hospital Cardiovascular Center, 1173 Arita, Miyazaki 880-2102, Japan

**Keywords:** Case report, Aortic regurgitation, Aortic valve commissure detachment, Localized aortic dissection, Transoesophageal echocardiography, Marfan syndrome

## Abstract

**Background:**

Aortic regurgitation (AR) associated with detachment of the aortic valve commissure is extremely rare. We present a case of progressively worsening severe chronic AR due to detachment of the aortic valve commissure during hospitalization that was confirmed with multimodality imaging.

**Case summary:**

A 50-year-old male with Marfan syndrome visited our hospital to receive treatment for cholelithiasis. Pre-operative examination revealed severe AR and aortic root aneurysm. Because the patient was asymptomatic, it was decided that cholecystectomy should be performed first. However, the patient’s heart failure worsened acutely when his blood pressure increased just before induction of anaesthesia. The patient required intubation and management of heart failure. Five days later, the patient underwent cholecystectomy. He was treated for heart failure and underwent open heart surgery on the 35th hospital day. Intraoperative transoesophageal echocardiography revealed that his AR was caused by both enlargement of the aortic root and localized dissection of the aortic valve commissure, which was supported by intraoperative findings and histopathological evaluation. Aortic regurgitation was exacerbated by a new localized dissection, resulting in acute worsening of heart failure.

**Discussion:**

Aortic valve commissure detachment can easily lead to sudden onset of severe AR, deteriorating haemodynamics, and acute pulmonary oedema. Since delayed medical treatment leads to poor clinical outcomes, prompt and accurate diagnosis and appropriately timed surgical intervention are essential. This very rare case of severe AR worsening due to spontaneous aortic valve commissure dissection was evaluated with multiple modalities during hospitalization. Understanding this clinical condition will help cardiologists provide better medical care.

**Learning points**

- Aortic valve commissure dissection is very difficult to diagnose and easily missed if not suspected.
- In addition to suspicion based on the clinical course, detailed assessment with transoesophageal echocardiography and computed tomography is essential.
- Accurate diagnosis is clinically important for considering the timing of surgery and the type of surgical procedure, such as valve-sparing root reconstruction.

## Introduction

Spontaneous detachment of the aortic valve commissure leading to acute aortic valve regurgitation (AR) is mostly due to blunt chest trauma, infective endocarditis, or ascending aortic dissection. Aortic regurgitation associated with detachment of the aortic valve commissure is extremely rare; there have only been a limited number of case reports.^1–10^ This condition has been described as aortic valve commissural detachment, dehiscence, avulsion, or tear. Although it is often difficult to definitively diagnose the cause of acute AR in the setting of acute heart failure before surgery, the development of transoesophageal echocardiography (TEE) and contrast-enhanced computed tomography (CT) with temporal and spatial resolution has made accurate diagnosis possible. We present a case of Marfan syndrome with progressively worsening severe chronic AR due to detachment of the aortic valve commissure during hospitalization that was confirmed on the basis of TEE, CT, intraoperative findings, and histopathological assessment.

## Summary figure

**Figure:**
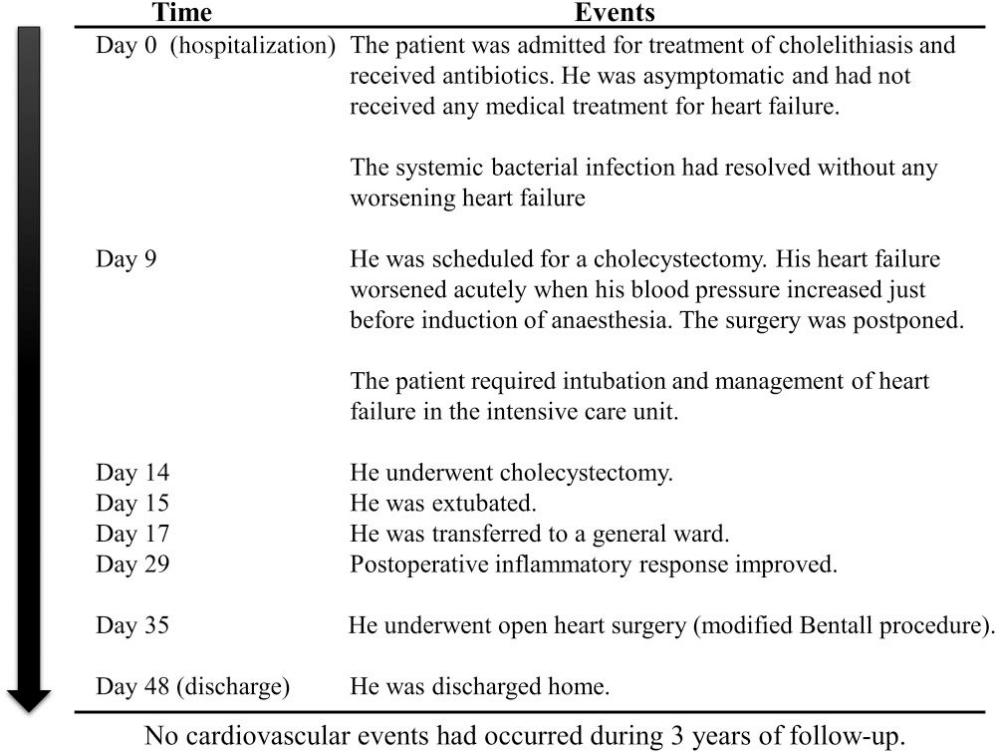


## Case presentation

**Figure 1:**
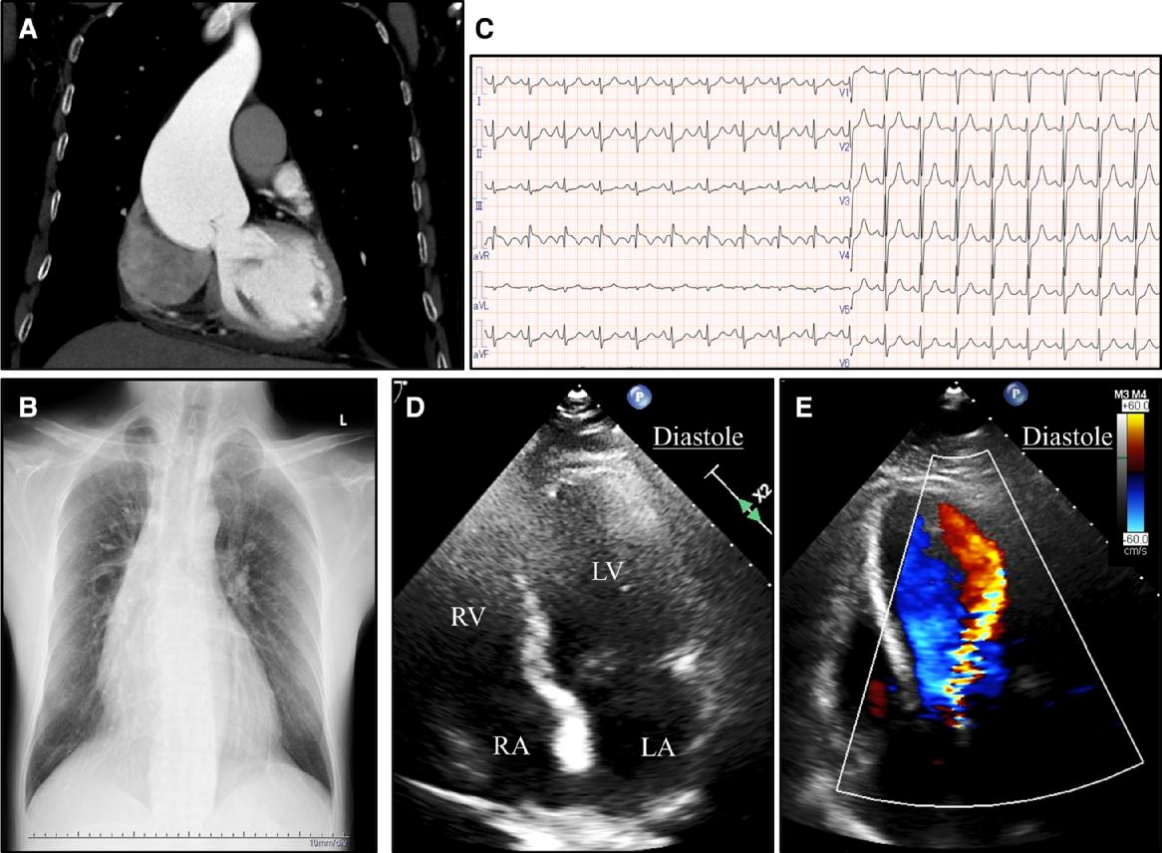
Assessment on admission. Contrast-enhanced computed tomography revealed an aortic root aneurysm (maximum diameter, 60 mm) but no evidence of dissection (*A*). Chest radiography showed mild pulmonary oedema but no pleural effusions and an enlarged cardiothoracic ratio (57.4%). Mild scoliosis was also observed (*B*). Electrocardiography showed sinus tachycardia (112 b.p.m.) and right axis deviation (*C*). Transthoracic echocardiography showed preserved left ventricular function (left ventricular ejection fraction, 68%), left ventricular enlargement (left ventricular end-diastolic dimension, 57 mm) (*D*), and severe aortic regurgitation (*E*). LA, left atrium; LV, left ventricle; RA, right atrium; RV, right ventricle.

A 50-year-old man presented to our hospital for treatment of cholelithiasis. Chest radiography revealed mediastinal enlargement, and cholecystectomy was being considered. Therefore, thoracoabdominal CT was performed in the arterial and venous phases to assess the course of the vessels. Pre-operative thoracoabdominal contrast-enhanced CT revealed an aortic root aneurysm with a maximum diameter of 60 mm (*Figure 1A*). The patient was evaluated pre-operatively by cardiologists and cardiovascular surgeons. He had no chest pain or signs of heart failure and was not receiving any medical treatment. He was 180 cm tall, weighed 68.4 kg, and had thoracic deformity and scoliosis (*Figure 1B*). The patient had no family history of Marfan syndrome. He had not undergone genetic testing and had no ectopia lentis but fulfilled the revised Ghent nosology for Marfan syndrome. He had aortic root aneurysm (*Z*-score, 8.17) and a systemic score of 11 points.^11^ Physical examination revealed a grade IV/VI to-and-fro murmur at the left sternal border (Erb’s point) without pitting oedema of the lower extremities. Pulse was 114 b.p.m. and regular. Blood pressure was 127/70 mmHg. Body temperature was 37.0°C. Respiratory rate was 20 breaths/minute and oxygen saturation was 95% on room air. Electrocardiography showed sinus tachycardia and right axis deviation (*Figure 1C*). Chest radiography showed mild pulmonary oedema but no pleural effusions, as well as an enlarged cardiothoracic ratio (*Figure 1C*). Although detailed evaluation was difficult because of pectus excavatum, transthoracic echocardiography (TTE) revealed severe AR with aortic root enlargement and left ventricular enlargement (left ventricular end-diastolic dimension, 57 mm) (*Figure 1D* and *E*). The AR jet was blowing into the left ventricle without extreme deviation. No obvious aortic valve prolapse was observed. Left ventricular systolic function was preserved with an ejection fraction of 68%. There was collapse of the inferior vena cava. There were no findings suggestive of pulmonary hypertension. Blood analysis showed elevated levels of hepatic and biliary enzymes, inflammatory response markers, and N-terminal pro-B–type natriuretic peptide (2380 pg/mL; normal range ≤ 125 pg/mL).

The patient had not been receiving medical treatment but was determined to have compensated heart failure. After discussion at a multidisciplinary conference, it was decided that AR and thoracic aortic aneurysm, which were found incidentally, should be further evaluated and treated after cholecystectomy. On the ninth day after admission, after the systemic bacterial infection had resolved without any worsening heart failure, the patient was scheduled for a cholecystectomy. However, while the patient was awake and undergoing lumbar anaesthesia, he experienced a sudden worsening of heart failure triggered by an increase in blood pressure due to mental and physical stress (168/137 mmHg). The surgery was postponed because the patient had to be intubated and treated for heart failure in the intensive care unit. Five days later, when his cardiac condition had improved, he underwent laparoscopic cholecystectomy. Post-operatively, the patient was clearly aware of unsteady gait and dyspnoea on exertion compared with the time of admission.

**Figure 2:**
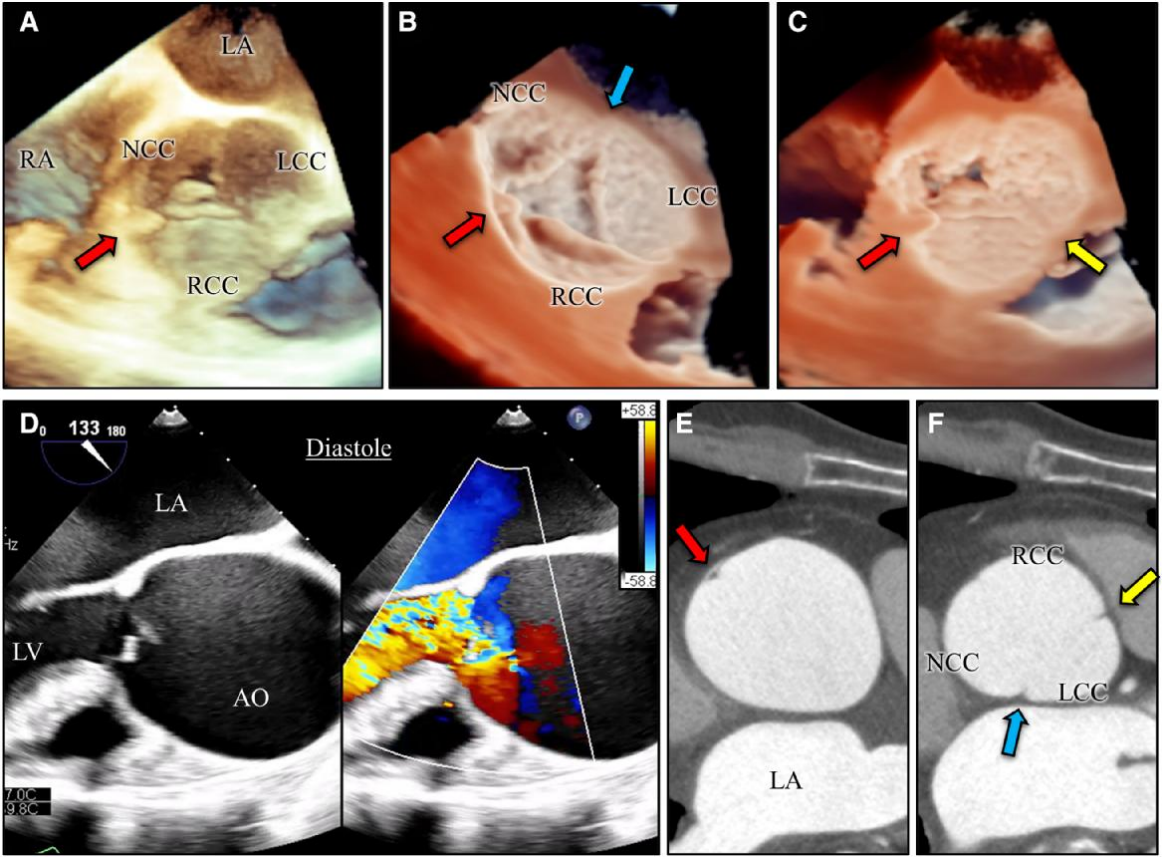
Imaging assessment of aortic valve commissure dissection. Intraoperative transoesophageal echocardiography revealed detachment of the aortic valve commissure between the right coronary cusp and non-coronary cusp (*A*–*C*, lower left arrows). No dissection was found in the other two aortic valve commissures (upper arrow and lower right arrow) (*B* and *C*). Severe aortic regurgitation associated with aortic root enlargement and prolapse of the non-coronary and right coronary cusps of the aortic valve were identified (*D*). Contrast-enhanced computed tomography revealed dissection of the aortic valve commissure from the aortic wall (upper left arrow), but there were no findings suggestive of dissection in the other commissures (lower arrow and upper right arrow) (*E* and *F*). AO, aorta; LA, left atrium; LCC, left coronary cusp; LV, left ventricle; NCC, non-coronary cusp; RA, right atrium; RCC, right coronary cusp.

**Figure 3:**
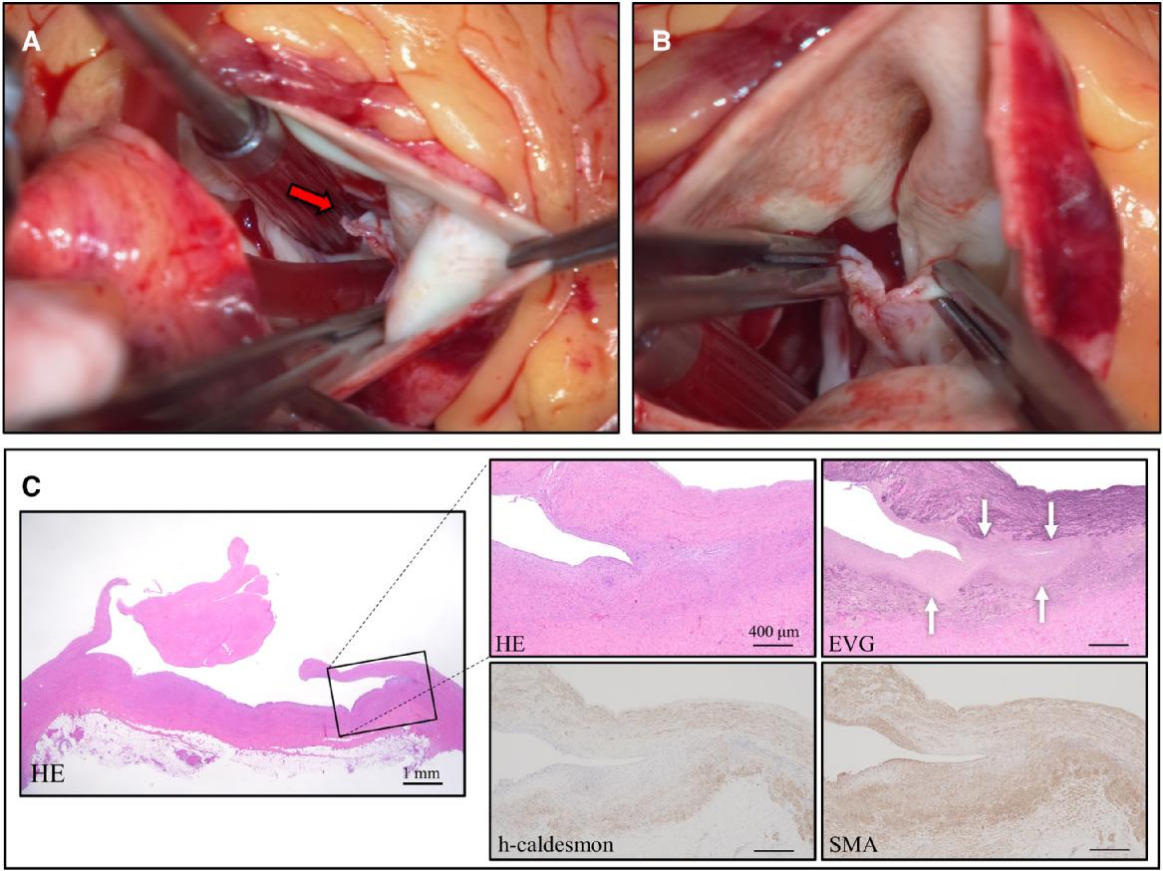
Intraoperative findings and histopathologic assessment. The aortic commissure detachment was captured with the surgeon’s camera (*A* and *B*). Histopathological assessment revealed cystic medial necrosis in the detached commissure (arrows) and the presence of smooth muscle actin–positive and h-caldesmon–negative smooth muscle cells, indicating that several weeks had passed since the detachment (*C*). EVG, Elastica van Gieson; HE, haematoxylin and eosin; SMA, smooth muscle actin.

After confirmation of the absence of a post-operative inflammatory response, the patient underwent cardiac CT. Cardiac surgery was performed 35 days after admission. Intraoperative TEE after induction of general anaesthesia revealed that the cause of AR included both enlargement of the aortic root and prolapse of the non-coronary and right coronary cusps of the aortic valve due to detachment of the aortic valve commissure between the right coronary cusp and non-coronary cusp (*Figure 2A–D*). Based on the TEE findings, we reviewed the contrast-enhanced CT images and confirmed dissection of the aortic valve commissure from the aortic wall, which was only visible in one slice (*Figure 2E* and *F*). We confirmed the commissural detachment and absence of other aortic dissections and infectious endocarditis intraoperatively (*Figure 3A* and *B*). The commissural dissection could not be identified before cardiac surgery; it was detected during a detailed intraoperative TEE assessment. Fortunately, the dissection did not extend into the ascending aorta. A modified Bentall procedure was performed as planned.

Additional histopathologic investigation of the surgical specimens revealed aortic root aneurysm and dissected commissure with cystic medial necrosis, which explained the fragility of the blood vessels (*Figure 3C*). Smooth muscle actin–positive and h-caldesmon–negative smooth muscle cells were found in the aortic valve commissural detachment (*Figure 3C*). These findings were consistent with the onset of the detachment being approximately 1 month prior to this operation. Based on the above findings, we concluded that AR was exacerbated by a new localized dissection associated with elevated blood pressure during lumbar anaesthesia, resulting in acute worsening of heart failure. The patient’s post-operative course was uneventful. No cardiovascular events had occurred during 3 years of follow-up.

## Discussion

Aortic regurgitation due to detachment of the aortic valve commissure usually occurs due to trauma, infective endocarditis, or ascending aortic dissection.^1^ Spontaneous commissure detachment is very rare but should be considered as a cause of AR. In this patient with an enlarged aortic root, increased tension on each coronary cusp and a fragile aortic wall due to cystic medial necrosis increased the possibility of aortic valve commissure dissection. Spontaneous aortic valve commissure detachment can easily lead to sudden onset of severe AR, deteriorating haemodynamics, and acute pulmonary oedema. As delayed medical treatment leads to poor clinical outcomes, prompt and accurate diagnosis and appropriately timed surgical intervention are essential.^1,2^ It is often difficult to manage heart failure medically in patients with severe AR. It should be noted that, as in the present case, the combination of two factors, aortic root enlargement and dissection of the commissure, might lead to progressive worsening of AR. The pathological examination clarified the timing of pathologic events in the present case. Hypertension is a risk factor for spontaneous aortic valve detachment.^6,10^ In the present case, induction of lumbar anaesthesia led to excessively high diastolic blood pressure, leading to commissural dissection and acute worsening of heart failure. Although several cases of successful aortic valve repair with commissural reconstruction have been reported,^3,8^ this patient had an enlarged aortic root; thus, commissural reconstruction alone was considered insufficient and intervention for the aortic root was added. Valve-sparing surgery was also considered; however, given the fragility of the aorta in patients with Marfan syndrome and previous experience with similar cases,^9,12^ the modified Bentall procedure was performed to ensure a better long-term outcome.

Transthoracic echocardiography allows for real-time observation of aortic valve leaflet movement and prolapse, enabling detailed investigation of the aetiology of AR. Sometimes, the precise mechanism of AR cannot be discerned with TTE because of unclear imaging due to obesity, pectus excavatum, or obstructive lung disease. Pre-operative diagnosis of aortic valve commissure detachment is difficult. Transthoracic echocardiography, which allows for the assessment of the aortic root from a closer position, provides more detailed views of the aortic valve and ascending aorta and is particularly useful in the diagnosis of localized dissection of the aortic valve commissure, which can be missed even with contrast-enhanced CT. High spatial and temporal resolution are the strengths of TEE. Three-dimensional (3D) TEE can also reveal differences by rendering each commissure in a 3D image, providing definitive imaging information for an accurate diagnosis. These informative images are very useful not only for echocardiologists and cardiovascular surgeons but also for information sharing among the cardiac team.

## Conclusion

This very rare case of severe AR worsening due to spontaneous aortic valve commissure dissection was evaluated with multiple modalities during hospitalization. It resulted in a good outcome with appropriate surgical intervention. Understanding this clinical condition will help cardiologists provide better medical care in the future.

## Data Availability

All data related to this case report are presented in the published manuscript.

## Lead author biography

Shun Nishino, MD, is a graduate of Hokkaido University, Japan. He currently works as a staff cardiologist at Miyazaki Medical Association Hospital Cardiovascular Center. He is specialized in echocardiography, especially in the field of valvular heart disease. His previous publications include clinical research works regarding ischaemic mitral regurgitation with 2D/3D echocardiography.

**Consent:** The authors confirm that written consent for the submission and publication of this case report, including images and associated text, has been obtained from the patient in accordance with the Committee on Publication Ethics (COPE) guidance.

**Funding**: None declared.
